# Impact of Nesting Socotra Cormorants on Terrestrial Invertebrate Communities

**DOI:** 10.3390/insects12070615

**Published:** 2021-07-07

**Authors:** Hiba Al Shehhi, Sabir Bin Muzaffar

**Affiliations:** 1 Department of Biology, United Arab Emirate University, Al Ain P.O. Box 15551, United Arab Emirates; hodarwish@moccae.gov.ae; 2 Marine Biodiversity Section, Ministry of Climate Change and Environment, Dubai P.O. Box 1509, United Arab Emirates; 3 National Water and Energy Center, United Arab Emirates University, Al Ain P.O. Box 15551, United Arab Emirates

**Keywords:** seabird, Socotra Cormorant, nesting, breeding, guano, terrestrial invertebrates

## Abstract

**Simple Summary:**

Seabirds that breed on remote islands often form dense nesting colonies. Nesting activities during the course of a breeding season could result in the deposition of large quantities of feces that could influence soil biodiversity. We determined the impact nesting activities of Socotra Cormorants on soil invertebrates using artificial substrate samplers. Nesting activities had variable effects on soil invertebrates. Isopods and spiders declined in due to nesting activities. Beetles and ants seemed to be unaffected by nesting activities. Ticks increased significantly but in non-nesting areas. Our study shows that seabird breeding activities determine long-term community structure of remote islands by affecting different invertebrate taxa in different ways.

**Abstract:**

Seabirds and some inland waterbirds nest in densely aggregated colonies. Nesting activities for a duration of months could lead to large quantities of guano deposition that affects the soil chemistry, flora and fauna. We assessed the effects of nesting Socotra Cormorants on soil invertebrates on Siniya Island, United Arab Emirates. Artificial substrate traps were set in nesting and non-nesting areas to sample invertebrates both before and after nesting had occurred. Diversity of soil invertebrate taxa decreased significantly in nesting areas compared to non-nesting areas after the commencement of nesting. This indicated that nesting activities had a negative effect on diversity. Among selected taxa, isopods and spiders decreased significantly in response to nesting activities. In contrast, ants were likely affected by habitat while beetles did not change significantly in response to nesting activities, suggesting that their numbers probably fluctuated in relation to seasonality. Ticks increased significantly but only in non-nesting areas. Thus, the impact of nesting varied between taxa depending on life history and seasonality. Our observations reflect the dynamic nature of invertebrate abundance that is affected by seasonality and the hyper-abundance of nesting seabirds.

## 1. Introduction

Seabirds and some inland waterbirds nest on islands or inaccessible cliffs on the mainland for a period of 2–6 months [1,2]. Nesting density of these birds is a robust indicator of their effects on plant communities, invertebrates and the nutrient content of the substrate [3,4]. Breeding seabirds transport substantial quantities of marine nutrients on to their terrestrial breeding colonies through the deposition of fecal material or guano, regurgitated food, carcasses and feathers [3,5]. Nutrients deposited on the land could influence the soil surface as well as subsurface layers where they penetrate, sometimes elevating potentially pathogenic species, such as *Escherichia coli* bacteria. In Chrzypsko Lake, Poland, a breeding population of 155–175 pairs of Great Cormorants (*Phalacrocorax nigrogularis*) caused an increase in the concentration of nitrogen and phosphorus in the ground water as well as an elevation in the *E. coli* and other coliform bacteria, within the boundaries of the colony [6]. Similarly, Socotra cormorants elevated concentrations of soil elements through allochthonous transport of nutrients on an island in the United Arab Emirates [7]. Offshore breeding sites with dense aggregations of cormorants or other seabirds have elevated soil nutrient content that regulates their plant and invertebrate assemblages [3]. Furthermore, breeding activities for extended periods often result in degradation of vegetation or the substrate [3,5]. For example, Great Cormorants that breed on islands in large temperate lakes cause a catastrophic decline in small shrubs and forest stands at the nesting areas [6]. Similarly, Double Crested Cormorants (*P. auritus*) breeding in forested islands of southeastern United States affected soil chemistry and tree health, although water chemistry and coliform abundance were not affected [4,8]. Long-term degradation in the quality of the habitat could cause breeding seabirds and other colonial waterbirds to seek alternative nesting sites, resulting in abandonment of their former breeding colonies [3,5].

Seabirds or inland waterbirds nesting in low densities likely add limited quantities of nutrients, such as nitrates and ammonium salts, compared to those nesting in large densities that could add huge quantities of guano and other materials [9]. Some of these nitrogenous wastes may be easily leached or desiccated [1,3,10]. On the other hand, phosphates could remain in soil for extended periods and may reflect long-term history of guano deposition of an island [11]. Altering the soil nitrogen and phosphorus ratios could limit or enhance plant growth depending on species-specific requirements, resulting in fundamental alteration of the terrestrial biota [12,13].

Seabirds can have complex effects on the community dynamics of plants and terrestrial invertebrates [14]. Differential uptake of nitrogen and phosphorus by plants at cormorant nesting sites is driven by variations in plant physiology and microclimate [12,15,16]. Herbivorous insects could be increased or decreased depending on plant diversity and biomass, ultimately affecting insect or other arthropod species composition [13,15,16,17]. For example, Lepidoptera larvae and aphids (herbivorous) occur in much higher abundance on plants in nesting areas that have vastly increased N and P content [15]. In comparison, herbivorous beetles have higher densities on islands that have high P content of soil and have been abandoned by breeding birds [15]. Elevated N and P content of the soil at nesting areas may not always translate into enhanced leaf nutrient content and therefore increased arthropod abundance [16]. For example, *Galerucella* (Coleoptera) numbers are high as long as the plants they fed on had high leaf N content and plant height, regardless of whether the soil N content was high [16]. In contrast, predatory groups, such as Hymenoptera, have high abundance at active breeding colonies as well as abandoned ones [15,18]. Abundance and species richness of predatory, scavenging and fungivorous beetles may be greatly increased on active cormorant breeding colonies [17]. In contrast, spiders and herbivorous beetles could be lower in abundance whereas chironomids and ticks (parasitizing seabirds) could be greatly increased in abundance due to high nest densities [17]. Furthermore, the effects of cormorant nesting on many plant species are toxic, allowing some plants to flourish only in areas not used by cormorants, with low N and P [16]. Thus, breeding activities and associated guano deposition could have complex effects on terrestrial communities of plants and invertebrates [19].

To this end, we studied the arthropod fauna from a large colony of Socotra Cormorants in the United Arab Emirates to (i) characterize soil invertebrates in a large seabird colony in UAE; and (ii) quantify the effect of nesting cormorants on invertebrate diversity and abundance.

## 2. Materials and Methods

### 2.1. Study Area and Species

The Socotra cormorant occurs in the Arabian Gulf and parts of the Gulf of Oman as well as extending as a separate population along the southern Omani shoreline into the Gulf of Aden [20]. The breeding population in the Arabian Gulf totals to about 97,150–123,150 breeding pairs nesting on 14–17 islands along the southern Arabian Gulf, with a large population occurring within the United Arab Emirates [21]. This study was conducted at Siniya island off the coast of Umm Al Quwain, UAE, which has a breeding population of 26,000–41,000 pairs of Socotra cormorants [22,23,24]. The habitat consists of desert shrubs (*Haloxylon*) scattered in patches across the island with sandy and open gravel plains.

### 2.2. Study Design

**Figure 1:**
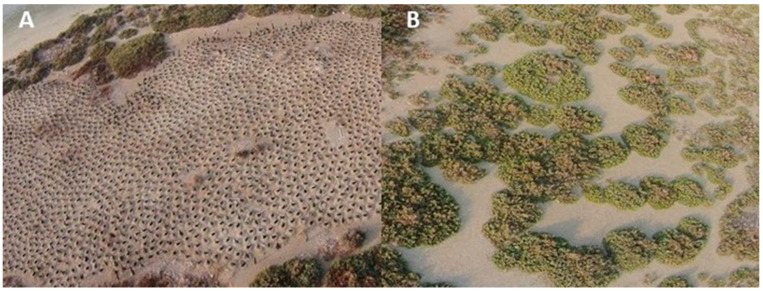
Aerial view of (**A**) nesting areas used by Socotra Cormorants showing individual nests (uniformly distributed dark shapes) in comparison with non-nesting areas (**B**) after nesting had occurred.

We constructed artificial substrate samplers from small rectangular plastic containers (15 cm × 8 cm × 7 cm) by adding a layer of loose sand from the surrounding habitat, drilling a small opening (2 cm diameter) on their side and partially burying them in the sand to a depth of 3 cm. We did not use standard pitfall traps because the environment is harsh, and the trapped invertebrate specimens as well as the fluids used to entrap them degrade quickly. We therefore chose to use the artificial substrate samplers that is frequented by invertebrates that remain alive at the time of collection. We recognize that these samplers could be selective, and we expect that some taxa (for example, certain beetle species and several ant species) may not be adequately collected due to differences in behavior or biology. However, since the same sampler was used in both nesting and non-nesting areas, we expect that the sampled invertebrate communities can be comparable to each other. The placement of the samples was arranged in a stratified, systematic design [25], with four 100 m transect lines spanning in nesting areas (designated ‘nesting area’) and four 100 m transect lines in areas not used for nesting (designated ‘non-nesting area’) by Socotra Cormorants in 2016 (Figure 1). One artificial substrate sampler was placed at each sampling point placed every 10 m along each 100 m transect, totaling to 10 sample points per transect line. Two transects in nesting areas and two transects in non-nesting areas were deployed from April to May were designated ‘before’ nesting. Similarly, samplers on two transects in nesting areas and two transects in non-nesting areas deployed from October to December and were designated ‘after’ nesting. Thus, altogether there were 80 sampling points (40 inside nesting areas and 40 in non-nesting areas). Of the 40 sampling points for each treatment (nesting or non-nesting), 20 were deployed before nesting and 20 were deployed after nesting had started. The samplers were placed in each sampling point for 30 days to allow colonization by arthropods. The samplers were lifted out of the sampling point after each sampling period by lifting the whole sampler and placing them in doubled freezer bags. Samples were transported within two hours of collection to the Entomology and Animal Ecology laboratory in the Biology Department at United Arab Emirates University and placed in freezers at –20 °C for future analysis.

### 2.3. Sorting, Enumeration and Statistical Analyses

The contents of each sample were filtered manually through a 2 mm mesh sieve to remove large visible pieces of debris. All arthropods were collected, categorized into taxonomic groups and counted. All collected arthropods were preserved in vials with 70% ethanol. All species were identified to the lowest possible taxonomic level using available references [26,27,28,29,30].

We calculated the Shannon–Wiener index (H) (Henceforth referred to as Shannon’s Index). Diversity of taxa was compared using the Diversity t test (Hutcheson’s *t*-test) [31] between nesting and non-nesting areas (to account for habitat effects) and before and after nesting had occurred (to account for the effect of nesting) [31]. Standard error for Shannon’s Index values were calculated using randomization methods (9999 replications) with replacement. Abundance of selected taxa (Isopoda, Araneae, Acari, Formicidae and Coleoptera) were designated response variables and nesting activities (before versus after nesting) and habitat (nesting versus non-nesting areas) were coded as explanatory variables to carry out two-way ANOVAs [31]. If interaction terms were significant, then it was inferred that habitat (nesting and non-nesting areas) had a significant effect on the abundance. When effect of habitat and the interaction term (nesting activities and habitat) were not significant, then the effects of nesting activities (before and after nesting) were tested separately using one-way ANOVAs [31]. In all cases, normality assumptions were tested by comparing residuals versus fits and the Shapiro–Wilk normality test. When assumptions of normality were not met, bootstrapping with 99,999 replications (built-in the software, [31]) were carried out to determine significance. All statistical analyses were preformed using PAST 3.20 (PAleontological STatistics) software [31] and significance level for α was set at 0.05.

## 3. Results

Terrestrial invertebrates totaling to 1560 specimens were collected (Table 1) from a total of 68 samples since 12 traps were lost. Thus, the individual sample sizes were 20 in nesting areas before nesting, 15 in nesting areas after nesting, 17 in non-nesting areas before nesting and 16 in non-nesting areas after nesting.

### 3.1. Diversity

**Figure 2:**
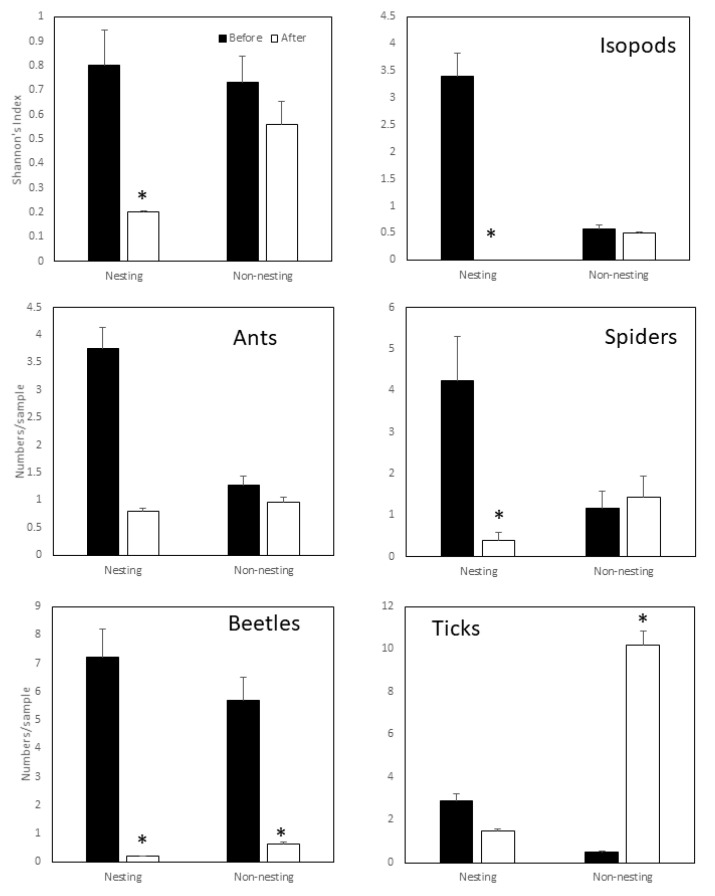
Comparison of the Shannon’s Index values and abundance (numbers per sample) of selected taxa in nesting and non-nesting areas both before and after nesting periods of Socotra Cormorants. Error bars indicate standard error. Standard error for Shannon’s Index values were calculated using randomization methods (9999 replications) with replacement. * represents differences that are significant at α = 0.05.

Mean Shannon’s diversity index decreased significantly in nesting areas after nesting had occurred (F = 73.14, df = 34.89, *p* =< 0.001) but no significant decrease was noted in non-nesting areas (F = 1.155, df = 37.99, *p* = 0.2893, Figure 2).

### 3.2. Changes in Selected Taxa

Isopoda, or isopods, were represented by two species, with *Armadillidium vulgare* being the dominant of the two species (Table 1). Two-way ANOVA showed that nesting activities (F*_nest_* = 11.10, df = 1, 64, *p* = 0.001; 99,999 replications), habitat type (F*_hab_* = 6.62, df = 1, 64, *p* = 0.01; 99,999 replications) and their interaction (F_nest*hab_ = 8.95, df = 1, 64, *p* = 0.004; 99,999 replications) were all highly significant. However, the number of isopods declined to zero in nesting areas after nesting had occurred but did not differ significantly in non-nesting areas during the same period (F = 0.04, df = 1, 32 *p* = 0.821; 99,999 replications Figure 2). Two-way ANOVA of the Hymenoptera, dominated by ants, showed that their abundance was not significantly related to nesting activities (F*_nest_* = 3.15, df = 2, 64, *p* = 0.081; 99,999 replications). However, habitat type (F*_hab_* = 4.30, df = 1, 64, *p* = 0.042; 99,999 replications) and the interaction between habitat and nesting activities (F_nest*hab_ = 4.38, df = 2, 64, *p* = 0.04; 99,999 replications) were both significantly related to abundance (Figure 2). Thus, there appeared to be no relationship between nesting activities and ant abundance and observed differences were likely due to differences in habitat type. Two-way ANOVA on spider abundance indicated that only nesting activities affected abundance significantly (F*_nest_* = 7.27, df = 1, 64, *p* = 0.008; 99,999 replications) but not habitat (F*_hab_* = 3.40, df = 1, 64, *p* = 0.06; 99,999 replications). Further one-way ANOVA showed that the decline in the number of spiders occurred consistently after nesting in nesting areas (F = 9.72, df = 1, 34, *p* = 0.0029; 99,999 replications) but the numbers in non-nesting areas remained unchanged (F = 0.16, df = 1, 32, *p* = 0.682, 99,999 replications). This suggested that the decline in spider numbers in the nesting areas occurred due to nesting activities. Two-way ANOVA on beetle abundance showed that nesting activities (F*_nest_* = 13.91, df = 2, 64, *p* = 0.00041; 99,999 replications) significantly affected beetle numbers but habitat type (F*_hab_* = 4.42, df = 1, 64, *p* = 0.05; 99,999 replications) and the interaction between nesting activities and habitat type (F_nest*hab_ = 3.36, df = 1, 64, *p* = 0.07; 99,999 replications) did not. Thus, we performed one-way ANOVA, which showed that beetles declined significantly in both nesting (F = 27.19, df = 1, 34, *p* = 0.00001; 99,999 replications) and non-nesting areas (F = 8.05, df = 1, 32, *p* = 0.002; 99,999 replications) (Figure 2). Two-way ANOVA on tick abundance showed that ticks (*Ornithodoros muesebecki*) were not affected by habitat type (F*_hab_* = 1.20, df = 1, 64, *p* = 0.27; 99,999 replications). Further one-way ANOVA showed that ticks remained unchanged in nesting areas (F = 0.25, df = 1, 34, *p* = 0.6249, 99,999 replications) but increased significantly in non-nesting areas after nesting (F = 2.83, df = 1, 32, *p* = 0.023; 99,999 replications, Figure 2).

## 4. Discussion

Many cormorant species come to offshore islands to breed and roost [2,21]. Seabirds venture into nearby feeding areas, returning to their nests, where they collectively deposit guano in large amounts [17,21]. Through their nest-building and defecation activities, seabirds strongly alter the terrestrial community structure of the breeding colonies [9,32,33,34]. The extent of the impact of seabirds on their nesting areas depends on density of nests as well as long-term, repeated use of the same sites [3]. Thus, large, densely aggregated colonies of breeding seabirds could deposit substantial quantities of guano resulting in long-term changes in nutrient content of the soil, often spanning over the course of a few decades [17]. The impact of such nutrient deposition is largely implied [3], and few studies have measured these effects quantitatively, e.g., [15,16,17]. The effects of nutrient enrichment on the diversity and abundance of terrestrial plants and invertebrates are variable [15]. The impact of cormorants on soil invertebrates has not been studied elsewhere. Here, we provide quantitative evidence on how cormorant nesting activities and associated guano deposition affect soil invertebrates in an arid environment.

Guano from Socotra cormorants become part of the soil and the impact on the vegetation is visible by the end of the season [7]. We found that Socotra cormorants significantly influence ground-dwelling invertebrate diversity and the abundance of selected taxa. Diversity of invertebrates declined in nesting areas after nesting had occurred, along with a decline in the abundance of selected taxa. The responses of selected taxa, however, did not show the same trend and changes occurred in both nesting and non-nesting areas. Arthropod communities show variable responses to nutrient enrichment [8,17]. For example, nesting activities of double-crested cormorants (*Phalacrocorax auritus*) on forested islands caused dramatic changes in the arthropod community from herbivores to mostly scavengers. In contrast, adjacent habitats that were not used for nesting did not show similar changes. Thus, high nutrient content could enhance selected species that then become dominant in the community, causing species diversity to fall [8].

*Armadillidium vulgare* was the most common isopod recorded in samples. Generally, nesting areas had less plant biomass compared to nesting areas. The decline in isopods in nesting areas was related to a combination of nesting and habitat factors. Plant biomass was low in nesting areas and this was lowered further due to guano deposited during the course of the season (not quantified). Collectively, this caused isopods to decrease in nesting areas. In comparison, the isopods in non-nesting areas were low in abundance before the nesting season and this did not change after nesting had occurred. Isopods are phyto-saprophagous and are abundant in moist places with decomposing vegetation. Seabirds nesting on the sub-Antarctic Adams Island, New Zealand, for example, caused an increase in plant growth due to nutrient enrichment (‘nutrient-trap effect’) from guano deposition [35]. This increased isopod and coleopteran abundance on sites enriched by nesting seabirds [35]. Although the habitat in our study area was markedly different, with a generally dry environment with scattered scrub vegetation, reductions of plant material caused by nesting and guano deposition could have caused the observed declines in isopods in nesting areas.

*Cataglyphis* is one of the most commonly occurring ant genus in arid regions, occurring in open areas [36]. The lack of a relationship between ant abundance and nesting activities indicated that these ants were more resistant to disturbance caused by breeding birds and the deposition of guano. Moreover, the ants in our study were probably more influenced by habitat factors suggesting that non-nesting areas and the associated plant cover represent more stable habitat for them. The most common spider taxa were the ‘ant spiders’ (family Zodariidae) that primarily feed on ants [36]. Guano deposition or disturbance from nesting birds did not affect spiders either. Other factors could be governing the abundance of ants and the ant spiders that feed on them.

Beetles are most abundant in areas influenced by seabirds, sometimes occurring in densities that are five times denser on nesting and roosting islands than on other islands [37]. Furthermore, densities inside nesting areas could be as much as six times higher compared to non-nesting areas [37]. In our study, beetles were found in high abundance in both nesting and non-nesting areas and their numbers declined in both. Darkling beetles (Coleoptera: Tenebrionidae) generally increased during the summer when their favored plant species thrive [38]. These plants provide moisture and refuge from extreme temperatures of the UAE summers (up to 50 °C in July [38]). Populations of many beetle species decline after summer as milder temperatures characterize the approach of the winter. Our observations of declines in beetle abundance before and after nesting in nesting and non-nesting areas is consistent with seasonal declines in beetles noted in other studies [38]. In addition, many studies document a shift from phytophagous, foraging invertebrate taxa to more saprophagous taxa (particularly in the abundance of carrion-feeding beetle species) [8,35]. In our study, only one specimen of carrion beetle (Silphidae) was found. Carrion beetles were commonly seen associated with carcasses of chicks that start to become abundant soon after the first eggs hatched (personal observation). This indicated that the samplers that we used were biased towards other species of actively foraging beetles and possibly were not good for sampling carrion beetles that were less mobile, gathering around and inside carcasses. Thus, a combination of sampling methods should be used to accurately assess the beetle density.

*Ornithodoros muesebecki* is a commonly occurring tick found on Siniya Island and other offshore islands in the Arabian Gulf [39]. High densities of ticks can occur on cormorant and other seabird breeding sites. Seabirds may avoid tick infestations during breeding, shifting nest site locations and even abandoning colonies [40]. Ticks in nesting areas did not significantly change after nesting. However, there was a marked increase in tick abundance in adjacent, non-nesting areas. This was contrary to expectation. *Ornithodoros muesebecki* are soft ticks (Acari: Argasidae) that feed on their hosts for brief periods (few minutes) and detach from the host to return to the substrate [16,39,40]. Socotra cormorant nests occur mostly in the open where they are exposed to the elements [22]. Ticks were not found in high densities on the cormorant nesting areas. However, ticks appear to aggregate in vegetated areas near the nesting sites [39]. Thus, our observation of a build-up of high tick numbers in adjacent non-nesting areas suggest that these areas serve as hiding places from where ticks could frequently launch attacks on nesting or nearby roosting birds.

We conclude that Socotra Cormorants have profound effects on soil invertebrate communities. Species diversity is reduced in nesting areas indicating community level changes. The impact on taxa was either positive or negative. Isopods and spiders declined in response to guano deposition. Beetles and ants were unaffected by nesting activities and their change in numbers were likely driven by seasonality. Ticks increased in non-nesting areas, suggesting other factors related to the microhabitat could be driving their numbers. Our observations reflect the dynamic nature of invertebrate abundance that is affected by seasonality and the hyper-abundance of nesting seabirds in this arid environment. The full extent of the interactions between plant communities, invertebrates and the influx of seabirds is not understood, and we suggest further studies to better understand these interactions.

## Figures and Tables

**Table 1:** Invertebrates collected from inside and outside nesting areas on Siniya Island, Umm Al Quwain, United Arab Emirates.

Inverbrate Taxa	Nesting	Non-Nesting
Isopoda		
*Armadillidium vulgare*	102	27
*Armadillidium album*	5	2
Arachnida: Acari		
*Ornithodoros muesebecki*	116	299
Unidentified mites	31	29
Arachnida: Araneae		
Zodariidae		
*Dusmadiores deserticola*	12	10
Pholcidae	2	0
Unidentified spiders	83	40
Insecta		
Orthoptera	1	0
Hemiptera	0	0
*Zelus*	1	2
Unidentified true bugs	0	1
Coleoptera		
Tenebrionidae		
*Eleodes*	20	6
*Gonocephalum missellum*	12	0
Carabidae		
*Scarites*	0	2
Chrysomelidae		
*Chrysolina grata*	0	2
*Chloropterus politus*	0	1
Anthicidae		
*Stricticollis modestus*	2	1
Anamorphidae		
*Symbiotes gibberosus*	24	3
Histeridae	1	5
Heteroceridae		
*Heterocerus harteni*	1	1
Silphidae	1	0
Meloidae		
*Nemognatha chrycomelina*	1	0
Unidentified beetles	177	175
Diptera		
*Anthomyia procellaris*	0	1
*Musca domestica*	6	0
Chironomidae	0	1
Chloropidae	5	14
Unidentified flies	3	2
Hymenoptera		
Formicidae		
*Cataglyphis arenarius*	19	10
*Cataglyphis viaticoides*	2	3
*Cataglyphis flavobrunneus*	3	9
*Cataglyphis adenensis*	109	36
*Crematogaster leaviusculs*	0	2
Bethylidae	1	0
Halictidae		
*Lasioglossum*	0	1
Unidentified 1 Hymenoptera (at least 4 spp)	2	4
Neuroptera	10	5

## Data Availability

The data is archived by the first author and may be provided if requested.

## References

[1] Schreiber E.A., Burger J. (2002). Biology of Marine Birds.

[2] Nelson J.B. (2005). Pelicans, Cormorants and Their Relatives: Pelecanidae, Sulidae, Phalacrocoracidae, Anhingidae, Fregatidae, Phaethontidae.

[3] Mulder C.P.H., Jones H.P., Kameda K., Palmborg C., Schmidt S., Ellis J.C., Vidal E. (2011). Impacts of seabirds on plant and soil properties. Seabird Islands.

[4] Dorr B.S., Fielder D.G. (2017). Double-crested cormorants: Too much of a good thing?. Fisheries.

[5] Stewart E.M., Michelutti N., Shenstone-Harris S., Grooms C., Weseloh C., Kimpe L.E., Smol J.P. (2015). Tracking the history and ecological changes of rising double-crested cormorant populations using pond sediments from islands in eastern Lake Ontario. PLoS ONE.

[6] Klimaszyk P. (2012). May a cormorant colony be a source of coliform and chemical pollution in a lake?. Oceanol. Hydrobiol. Stud..

[7] Ksiksi T.S., Muzaffar S.B., Gubiani R., Alshihi R.M. (2015). The impact of nesting Socotra cormorants on soil chemistry and vegetation in a large colony in the United Arab Emirates. Diversity.

[8] Lafferty D.J., Hanson-Dorr K.C., Prisock A.M., Dorr B.S. (2016). Biotic and abiotic impacts of double-crested cormorant breeding colonies on forested islands in the southeastern United States. For. Ecol. Manage..

[9] Ellis J.C., Fariña J.M., Witman J.D. (2006). Nutrient transfer from sea to land: The case of gulls and cormorants in the Gulf of Maine. J. Anim. Ecol..

[10] Wait D., Aubrey D., Anderson W. (2005). Seabird guano influences on desert islands: Soil chemistry and herbaceous species richness and productivity. J. Arid. Environ..

[11] Sun L., Xie Z., Zhao J. (2000). Palaeoecology: A 3000-year record of penguin populations. Nature.

[12] Rajakaruna N., Pope N., Perez-Orozco J., Harris T.B. (2009). Ornithocoprophilous plants of Mount Desert Rock, a remote bird-nesting island in the Gulf of Maine, USA. Rhodora.

[13] Kolb G., Palmborg C., Hambäck P.A. (2013). Ecological stoichiometry and density responses of plant-arthropod communities on cormorant nesting islands. PLoS ONE.

[14] Gagnon K., Sjöroos J., Yli-Rosti J., Stark M., Rothäusler E., Jormalainen V. (2016). Nutrient enrichment overwhelms top-down control in algal communities around cormorant colonies. J. Exp. Mar. Biol. Ecol..

[15] Kolb G.S., Jerling L., Hambäck P.A. (2010). The impact of cormorants on plant-arthropod food webs on their nesting islands. Ecosystems.

[16] Kolb G., Hambäck P.A. (2015). Dynamic responses in a plant-insect system to fertilization by cormorant feces. Insects.

[17] Kolb G.S., Jerling L., Essenberg C., Palmborg C., Hambäck P.A. (2012). The impact of nesting cormorants on plant and arthropod diversity. Ecography.

[18] Kagata H., Ohgushi T. (2006). Nitrogen homeostasis in a willow leaf beetle, *Plagiodera versicolora*, is independent of host plant quality. Entomol. Exp. Appl..

[19] Gratton C., Denno R.F. (2003). Seasonal shift from bottom-up to top-down impact in phytophagous insect populations. Oecologia.

[20] Jennings M.C. (2010). Atlas of the breeding birds of Arabia. Fauna of Arabia.

[21] Muzaffar S.B. (2020). Seabirds in the Arabian Gulf: Ecology, movements and conservation. J. Aquat. Ecosyst. Health Manage..

[22] Muzaffar S.B., Gubiani R., Benjamin S. (2020). Reproductive Performance of the Socotra Cormorant *Phalacrocorax nigrogularis* on Siniya Island, United Arab Emirates: Planted Trees Increase Hatching Success. Waterbirds.

[23] Muzaffar S.B., Whelan R., Clarke C., Gubiani R., Benjamin S. (2017). Breeding Population Biology in Socotra Cormorants (*Phalacrocorax nigrogularis*) in the United Arab Emirates. Waterbirds.

[24] King H. (2004). Communal behaviour of Socotra Cormorant, Bahrain. Phoenix.

[25] Sokal R.R., Rohlf F.J. (2012). Biometry: The Principles and Practice of Statistics in Biological Research.

[26] Van Harten A. (2008). Arthropod Fauna of the United Arab Emirates.

[27] Van Harten A. (2009). Arthropod Fauna of the United Arab Emirates.

[28] Van Harten A. (2010). Arthropod Fauna of the United Arab Emirates.

[29] Van Harten A. (2011). Arthropod Fauna of the United Arab Emirates.

[30] Van Harten A. (2014). Arthropod Fauna of the United Arab Emirates.

[31] Hammer O. (2018). Paleontological Statistics.

[32] Wootton J.T. (1991). Direct and indirect effects of nutrients on intertidal community structure—Variable consequences of seabird guano. J. Exp. Mar. Biol. Ecol..

[33] Polis G.A., Hurd S.D. (1995). Extraordinarily high spider densities on islands: Flow of energy from the marine to terrestrial food webs and the absence of predation. Proc. Natl. Acad. Sci. USA.

[34] Anderson W.B., Polis G.A. (1999). Nutrient fluxes from water to land: Seabirds affect plant nutrient status on Gulf of California islands. Oecologia.

[35] Bassett I.E., Elliott G.P., Walker K.J., Thorpe S., Beggs J.R. (2014). Are nesting seabirds important determinants of invertebrate community composition on sub-Antarctic Adams Island?. Polar. Biol..

[36] Collingwood C.A., Agosti D., Sharaf M.R., van Harten A. (2011). Order Hymenoptera, family Formicidae. Arthropod Fauna UAE.

[37] Sánchez-Piñero F.L., Polis G.A. (2000). Bottom-up dynamics of allochthonous input: Direct and indirect effects of seabirds on islands. Ecology.

[38] Saji A., Al-Dhaheri S.S. (2011). Ecological distribution and seasonality of darkling beetles (Coleoptera: Tenebrionidae) in the Western Region of Abu Dhabi, UAE. Middle East J. Sci. Res..

[39] Al-Deeb M., Frangoulidis D., Walter M., Koempf D., Fischer S., Petney T., Muzaffar S.B. (2015). Coxiella-like endosymbiont in argasid ticks (*Ornithodoros muesebecki*) from a Socotra Cormorant colony in Umm Al Quwain, United Arab Emirates. Ticks Tick-borne Dis..

[40] Feare C.J., Gill E.L. (1997). The life cycle of the tick Amblyomma loculosum in sooty tern Sterna fuscata colonies in the Seychelles. J. Zool. Lond..

